# Robustness to extinction and plasticity derived from mutualistic bipartite ecological networks

**DOI:** 10.1038/s41598-020-66131-5

**Published:** 2020-06-17

**Authors:** Somaye Sheykhali, Juan Fernández-Gracia, Anna Traveset, Maren Ziegler, Christian R. Voolstra, Carlos M. Duarte, Víctor M. Eguíluz

**Affiliations:** 1 Instituto de Física Interdisciplinar y Sistemas Complejos IFISC (CSIC-UIB), Palma de Mallorca, E-07122 Spain; 2 Instituto Mediterráneo de Estudios Avanzados IMEDEA (CSIC-UIB), E07121 Esporles, Spain; 3 Department of Animal Ecology & Systematics, Justus Liebig University, Heinrich-Buff-Ring 26-32 IFZ, 35392 Giessen, Germany; 4 Department of Biology, University of Konstanz, Konstanz, 78457 Germany; 5 Red Sea Research Center, King Abdullah University of Science and Technology, Thuwal, Kingdom of Saudi Arabia

**Keywords:** Ecological networks, Biodiversity, Complex networks

## Abstract

Understanding the response of ecological networks to perturbations and disruptive events is needed to anticipate the biodiversity loss and extinction cascades. Here, we study how network plasticity reshapes the topology of mutualistic networks in response to species loss. We analyze more than one hundred empirical mutualistic networks and considered random and targeted removal as mechanisms of species extinction. Network plasticity is modeled as either random rewiring, as the most parsimonious approach, or resource affinity-driven rewiring, as a proxy for encoding the phylogenetic similarity and functional redundancy among species. This redundancy should be positively correlated with the robustness of an ecosystem, as functions can be taken by other species once one of them is extinct. We show that effective modularity, *i*.*e*. the ability of an ecosystem to adapt or restructure, increases with increasing numbers of extinctions, and with decreasing the replacement probability. Importantly, modularity is mostly affected by the extinction rather than by rewiring mechanisms. These changes in community structure are reflected in the robustness and stability due to their positive correlation with modularity. Resource affinity-driven rewiring offers an increase of modularity, robustness, and stability which could be an evolutionary favored mechanism to prevent a cascade of co-extinctions.

## Introduction

Network adaptation reflects the ability of a system to respond to external (e.g., environmental) perturbations in terms of establishing dynamic interactions between the remaining elements. This sort of adaptation based on the coevolution of topologies and states appears in many network systems^1,2^, including opinion formation^3,4^, the spread of infectious diseases^5^, online social networks^6^, ecological and biological networks^7–11^, power grids^12^ and chemistry^13^. An important class of adaptation dynamics occurs as a consequence of extinction events altering network structure. Examples include the disappearance of companies and technologies due to innovations and market competition^14,15^, neurons extinction^16^, gene extinction^17^, extinction of the least populated molecular species^18^ local extinction of species in biological networks due to perturbation such as pollution or human disturbance^19,20^, habitat loss^21^, climate change^22,23^ or extinction of interaction partners^24,25^.

In ecosystems, loss or declining abundance of one species can cause the extinction of other dependent species^26–28^. Understanding how ecosystems respond to species loss has hence been the subject of numerous theoretical and experimental studies^29,30^. While some communities exhibit density compensation^31,32^, a common form of adaptation in food webs is represented by the rewiring of feeding links among members of species in response to variation in resources^33–37^. Functional redundancy can help in the process of rewiring, as species have the ability to replace each other. So, for example, pollination networks have been shown to be fairly robust against species extinctions as a result of bipartite network asymmetry (redundancy in the number of floral visitors per plant^25^). However, trophic rewiring can lead to overexploitation of resources and aggravate the effects of species loss on food webs^38^.

From the perspective of the topological structure of the interactions of the species that compose ecosystems, recent studies have revealed key structural features which are believed to influence ecological dynamics^39^: robustness, defined in terms of the largest connected component^40^; nestedness, where specialists interact with a subset of the whole set of species that generalists interact with^41^; modularity, which refers to densely connected non-overlapping subsets of species (modules) with weak interactions between them^42,43^; and stability, which can be measured as the largest eigenvalue of the appropriate matrix^44^.

Some studies have analyzed these characteristics as the product of a temporal evolutionary process^45,46^. Based on previous results, modular structure in species interactions of mutualistic networks hinders the loss of species and promotes the long term persistence of ecological communities, as the compartments buffer the propagation of extinctions across the network^47–51^. More recent research provided empirical evidence to test this hypothesis experimentally and confirmed that a modular network structure enhances species persistence^52^. Here we used a dynamical modeling approach to investigate how different extinction-induced rewiring scenarios modify the structure of ecological bipartite networks. In particular, we investigated the effects on topological robustness, measured as the degree of connectivity within the network; network modularity, seen as the compartmentalized nature of the networks, and finally, dynamical stability, measured through the change in the largest eigenvalue of the adjacency matrix.

## Materials and Methods

### Bipartite networks from empirical mutualistic networks

We analyzed 130 empirical mutualistic bipartite networks consisting of 101 plant-pollinator, 25 seed-dispersal, 3 plant-ant, and 1 host-symbiont association (see Table S1, data available in the Web of Life database^53^ and^54,55^). We considered here only the presence or absence of the interaction, *i*.*e*. unweighted undirected bipartite networks.

Bipartite networks contain two types of nodes: resources and consumers. Resources represent plants (or flowers, seeds, hosts) while consumers represent pollinators (or dispersers, symbionts). They contain, thus, *n* resources and *m* consumers, and the adjacency matrix *A*(*n* + *m*, *n* + *m*) captures the pairwise interactions between resources and consumers: *A*_*ij*_ = 1 if there is a pairwise interaction between resource *i* and consumer *j*, and *A*_*ij*_ = 0 otherwise. Thus, the adjacency matrix can be written as

1 $$A=[\begin{array}{cc}{{\bf{0}}}_{n\times n} & {\tilde{A}}_{n\times m}\\ {({\tilde{A}}^{T})}_{m\times n} & {{\bf{0}}}_{m\times m}\end{array}]$$

where *Ã* is the incidence matrix, *T* indicates the matrix transpose and **0**_*m*×*m*_ is the all-zero matrix and the subindices indicate the number of rows and columns.

### Extinction-adaptation modeling

At each time step, the dynamics proceeds as follows:

1. *Extinction*: a resource node is removed.
2. *Adaptation*: each link that was attached to the extinct resource node is rewired to a different resource node with probability *r*.

The rewiring probability *r* varies between 0 and 1, where *r* = 0 represents the no-rewiring case and *r* = 1 the case where each link is attempted to be rewired.

We explored two scenarios for extinction: (i) random extinction, selecting the resource for extinction at random from the available ones; and (ii) directed extinction, selecting the resource proportionally to its degree, *i*.*e*. the number of consumers interacting with it. We considered again two adaptation scenarios, in both cases the consumer is maintained while the resource is rewired: (i) random rewiring, where the extinct resource is replaced by an existing resource at random; and (ii) resource affinity-driven rewiring, where the extinct resource is replaced by another resource selected proportionally to the number of common consumers shared with it. Thus, for instance, in the case of a plant-pollination network, an extinct plant species would be replaced with the largest probability by another species sharing the highest number of pollinators with the extinct one, which is likely to be the phylogenetically most closely related or ecologically redundant species. Hence, if the resource *j* is selected for extinction, the number of shared consumers with resource *k* is given by:

2 $$O(kj)=|{E}_{j}\cap {E}_{k}|$$

where *E*_*j*_ and *E*_*k*_ are the set of consumers connected with resource *j* and *k*. Thus, the probability that resource *k* is selected as replacement of resource *j* by resource affinity-driven rewiring is given by

3 $${p}_{r}(k;j)=\frac{O(kj)}{{\sum }_{l}\,O(lj)}.$$

**Figure 1:**
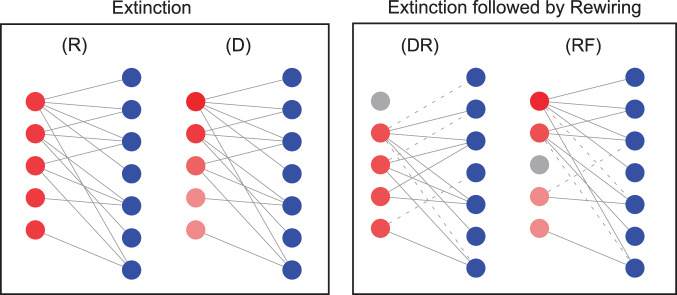
Schematic extinction and rewiring scenarios of a bipartite graph. Node colors represent resources/consumer species: red nodes represent resources, blue ones represent consumers and grey nodes represent extinct nodes. Solid lines characterize interactions among consumers and resources and dashed lines characterize rewired interactions. Extinction scenarios: consumers lose their preferred resource by (R) random extinctions and (D) directed extinctions; Rewiring scenarios: (DR) directed extinction followed by random rewiring, and (RF) random extinction followed by resource affinity rewiring. The probability of extinction (left) or rewiring (right) of the resource nodes is encoded in the intensity of the color of the resource nodes (darker for higher probability), consumers have the same color because they are not actively rewired in our model.

If the new link already existed, no new link is added. In total, we explored four extinction-adaptation scenarios: RR, random extinction and random adaptation, RF, random extinction and resource affinity-driven adaptation, DR, directed extinction and random adaptation, and DF, directed extinction and resource affinity-driven adaptation (Fig. 1).

### Robustness

Due to the extinctions, the bipartite networks eventually break up in several components. The relative size of the largest connected component in the network after a fraction f of resource nodes are extinct, *S*(*f*), reflects the ability of the network to maintain itself connected^40,56^. The behavior of the relative size of the largest component can be summarized in the robustness *ρ*, measured as the area under the *S*(*f*) curve:

4 $$\rho =\frac{1}{n}\,\mathop{\sum }\limits_{i=0}^{n}\,S(i/n).$$

A value of *R* close to one means that as nodes are being removed the network does not fragment, consisting of a single component all the way; while a value of *ρ* close to zero signals that the network fragments very easily into small disconnected components.

### Modularity

Modularity measures the presence of densely connected non-overlapping subsets of nodes called communities or modules. For bipartite networks, the modularity can be measured using Barber’s expansion of modularity with specific constraints reflected in the null model^57^ as

5 $$Q=\frac{1}{L}\,\mathop{\sum }\limits_{i=1}^{n}\,\mathop{\sum }\limits_{j=1}^{m}\,({\tilde{A}}_{ij}-{B}_{ij})\,\delta ({g}_{i},{h}_{j})=\frac{1}{L}\,\mathop{\sum }\limits_{i=1}^{n}\,\mathop{\sum }\limits_{j=1}^{m}\,\left({\tilde{A}}_{ij}-\frac{{k}_{i}{d}_{j}}{L}\right)\,\delta ({g}_{i},{h}_{j})$$

where *g*_*i*_ and *h*_*j*_ are the community indices that resources *i* and consumers *j* are assigned to, and *L* is the number of links in the network. The expected probability of interactions between two types of nodes is given by matrix *B*. Based on the null model, that considers random pairings between two classes of nodes with given degree sequences, the *B*_*ij*_ elements are proportional to the degree of resource *k*_*i*_ and the degree of consumer *d*_*j*_ in the original network $\frac{{k}_{i}{d}_{j}}{L}$. We have used the open-source library BiMat^58^ to find the community structure that maximizes Eq. (5). To test whether the empirical networks are significantly more modular than random ones, we calculated the modularity of each empirical network and compared them with the modularity of 1000 randomized networks with the same species degree distribution as the empirical network.

The sequence of extinction-rewiring events eventually breaks the bipartite networks in several components. To correct for the network breakout of the bipartite network, we introduced the effective modularity *Q*_*e*_, measured as the product of the modularity and the relative size of the largest connected component (fraction of nodes in the largest connected component): *Q*_*e*_ = *SQ*.

The changes in the community structure of bipartite networks can be quantified with the module persistence Π_*ij*_ defined as the probability that two nodes remain in the same community if they were initially in the same community, Π_*ij*_(*g*_*i*_ = *h*_*j*_, *t*|*g*_*i*_ = *h*_*j*_, *t*_0_), where *g*_*i*_ (*h*_*j*_) is the community to which node *i* (*j*) belongs.

### Stability

To study the relationship between the structure and the stability of an ecosystem, May considered positive and negative interactions to represent the relations among species in an ecological community and analyzed the eigenvalues of the adjacency matrix as a measure of the stability^44^. The assumption is that the adjacency matrix captures the linear stability of the dynamics of the ecosystem. In our case, the interaction is given by a matrix that reflects the bipartite structure and the mutualistic interactions, that is, all the entries are non-negative. In general, if the dynamics is given by a local dynamics and an interaction term:

6 $$\begin{array}{rcl}\frac{d{x}_{i}}{dt} & = & {{\mathscr{L}}}_{i}(\overrightarrow{x})\\  & = & f[{x}_{i}]+k\,\sum _{j}\,{A}_{ij}{x}_{j}\end{array}$$

where *x*_*i*_ is the abundance of species *i*, *f*(*x*_*i*_) are the local dynamics, *k* is a coupling strength and *A*_*ij*_ the adjacency matrix and therefore we only take into account positive interactions (mutualistic). The stability of this system around a fixed point ${\overrightarrow{x}}^{\ast }$ can be analyzed by evaluating the largest eigenvalue of the Jacobian matrix ${J}_{ij}=\frac{\partial {{\mathscr{L}}}_{i}(\overrightarrow{x})}{\partial {x}_{j}}=\frac{f({x}_{i})}{\partial {x}_{j}}+k{A}_{ij}$ at the feasible equilibrium point ($\overrightarrow{x}={\overrightarrow{x}}^{\ast }$), for which the off-diagonal elements coincide with *kA*_*ij*_. If the local dynamics are the same for all the elements, the diagonal elements are all the same and thus only contribute to the largest eigenvalue as an offset. If all the eigenvalues have a negative real part, then the equilibrium is stable and the perturbed system will go back to the equilibrium, but if any of the eigenvalues have a positive real part, the perturbed system will move away from the equilibrium. Therefore the eigenvalues of the Jacobian can be analyzed in terms of the eigenvalues of the adjacency matrix^59^.

We measured the change in stability of a mutualistic bipartite network as the relative change of the leading eigenvalue, *λ*, of the (*n* + *m*) × (*n* + *m*) adjacency matrix as extinction-adaptation events occur, with respect to the leading eigenvalue of the original matrix, *λ*_0_:

7 $$\Sigma =\frac{{\lambda }_{0}-\lambda }{{\lambda }_{0}}.$$

Note that the sign reflects an increase (positive) or a decrease (negative) of the stability, *i*.*e*., a lower (higher) eigenvalue. In general, we are interested in expected values

8 $$\langle \Sigma \rangle =\frac{{\lambda }_{0}-\langle \lambda \rangle }{{\lambda }_{0}},$$

where 〈·〉 denotes an average over realizations of the extinction-adaptation dynamics.

## Results

For each bipartite network analyzed, we reported the results of 100 realizations of the dynamics (unless otherwise indicated) for each scenario. For the sake of clarity, we show results for the five largest networks of different mutualistic interaction types: plant-pollinator (two of them), seed-dispersal, host-symbiont, and plant-ant (Table 1). The results for all the 130 networks are available in the Supporting Information (see Table S1).

**Table 1:** Sample mutualistic ecological bipartite networks.

Ecosystem	Species interactions	N	Interactions
Andean scrub^91^	Plants	87	372
	Pollinators	98	
Montane forest^92^	Plants	96	923
	Pollinators	276	
Coral Reef^54,55^	Host	92	1931
	Symbiont	693	
Atlantic Forest^93^	Seed	207	1121
	Dispersal	110	
Rain forest^56^	Plants	51	923
	Ants	41	

The table shows basic quantities of the five bipartite networks exemplifying different types of mutualistic relations such as plants and pollinators.

### Robustness

**Figure 2:**
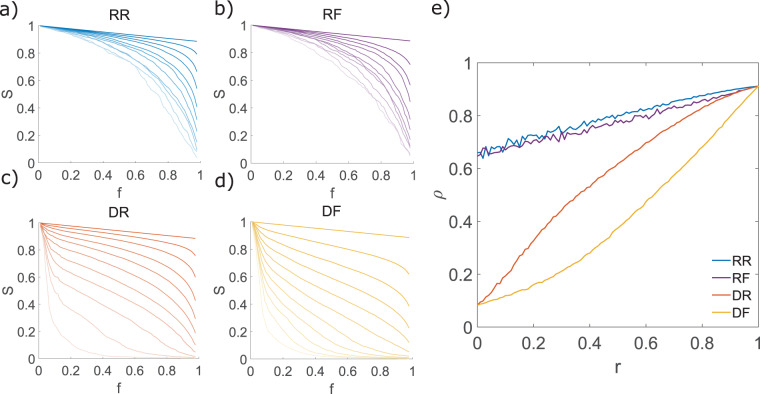
Robustness (*ρ*) and the size of the largest connected component *S* of the Coral Reef network for the different extinction and adaptation scenarios. (**a**–**d**) Size of the largest connected component as a function of the fraction of extinction events *f* and different rewiring probabilities $r\in [0,1]$ (lighter color refers to lower rewiring probability *r*). (**e**) Robustness (*ρ*) as a function of rewiring links probability for the different scenarios. Reported values are averaged over 1000 realizations of the extinction-adaptation sequences.

For our model, the breakup of the networks depended mostly on the extinction scenario, with a sharp decrease in size of the largest component with a few extinct resources in the directed scenario contrasting with a smoother decay in the random scenario (Fig. 2). Furthermore, for random extinctions, the rewiring mechanism had very little influence on the robustness, while for directed extinctions resource affinity-driven rewiring was more detrimental than random rewiring. The results for the rest of the networks were qualitatively similar (see Fig. S1 in the Supporting Information).

### Modularity

**Figure 3:**
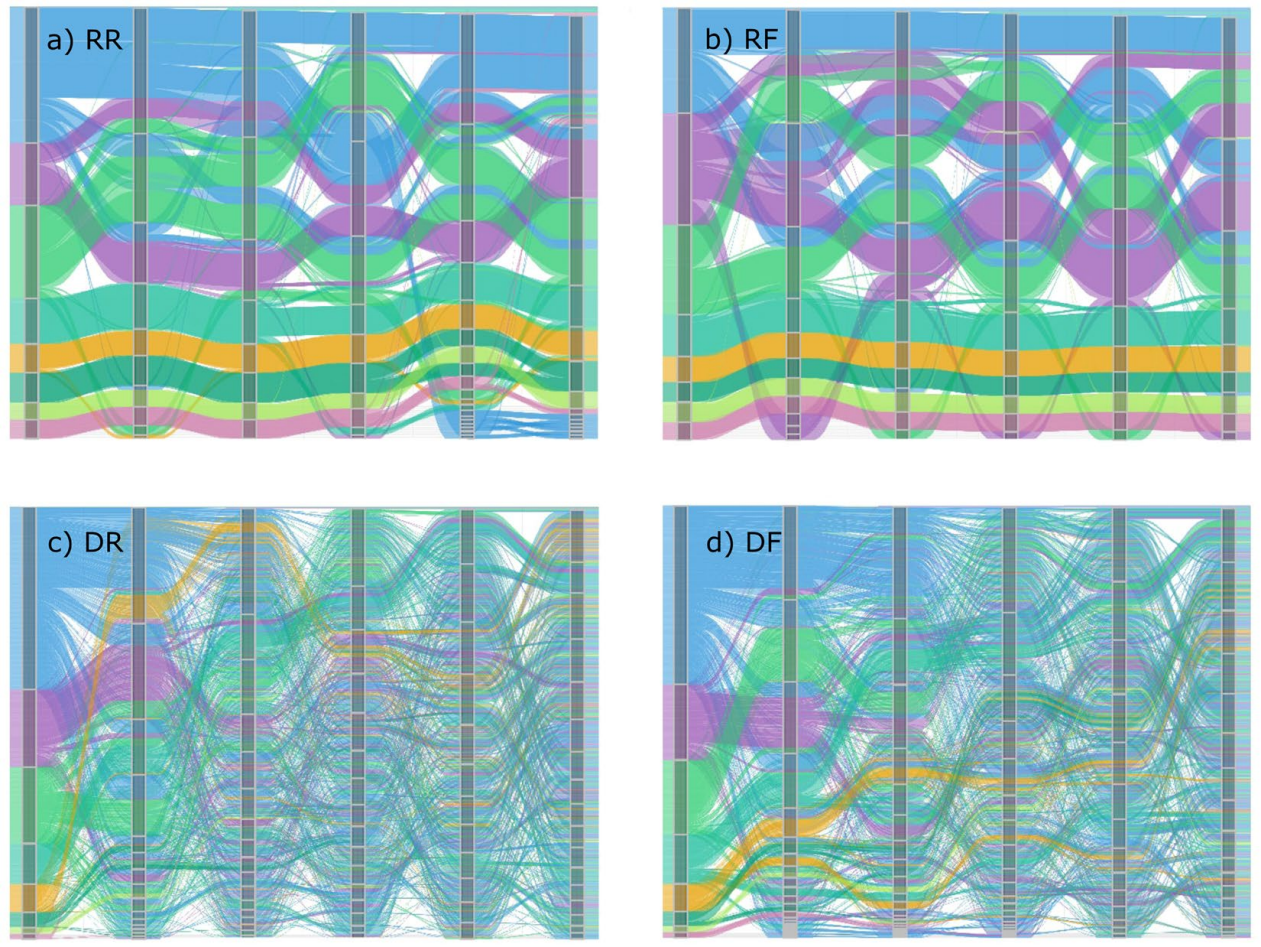
Transformation of the coral microalgal community structure over time (Table 1) after each extinction-adaptation event: (**a**) RR, Random species extinction - Random links adaptation; (**b**) RF, Random species extinction - Resource Affinity links adaptation; (**c**) DR, Directed species extinction - Random links adaptation; and (**d**) DF, Directed species extinction - Resource Affinity links adaptation. Each of the colored streams represents a species in the network. The columns formed by grey boxes stand for moments between extinction-adaptation events and the boxes encapsulate the species in the same community. The color of each species matches to which community they belonged initially. For each step the communities are ordered so that crossings among streams are minimized. The rewiring probability is *r* = 1.

The transformation of the modular structure over time with extinction-adaptation events shows that the removal of nodes and rewiring of links produce changes that affect the global organization of the mutualistic networks, as it can be qualitatively observed in the alluvial maps shown in Fig. 3. To quantify the reorganization of the networks, we measured how the modularity changes with rewiring probability and the fraction of nodes removed.

**Figure 4:**
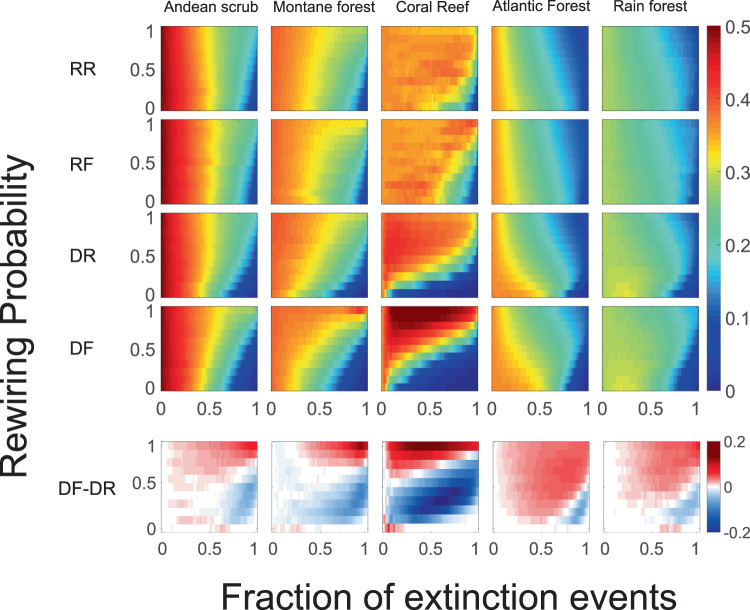
Effective modularity for the different extinction-adaptation scenarios. (Top) The effective modularity, where the modularity is defined as Eq. (5), is represented in a color scale from *Q*_*e*_ = 0 (blue) to *Q*_*e*_ = 0.5 (red), in each panel the x-axis is the fraction of extinction events and the y-axis the rewiring probability *r*. Each column displays the results for each data set. From left to right: Andean scrub, Montane forest, Coral Reef, Atlantic Forest, Rain forest (see Table 1); each row corresponds to the four extinction-adaptation dynamics. From top to bottom: RR, random extinction-random rewiring, RF, random extinction-resource affinity driven rewiring, DR, directed extinction-random rewiring, and DF, directed extinction-resource affinity driven rewiring. (Bottom) The difference of the effective modularity for DF and DR for the same ecosystem. Results are averaged over 100 realizations of the extinction-adaptation sequences for the different scenarios.

The effective modularity shows an idiosyncratic effect of the extinction-adaptation, in the sense that it depends mostly on the network analyzed and not on the specific extinction/rewiring scenario. In one extreme, bipartite networks show an increase in *Q*_*e*_ with rewiring. This is the case for the Coral Reef and Montane Forest networks (top panel of Fig. 4) and 88% (89/101) of the plant-pollinator and 60% (15/25) plant-seed dispersal networks (see Figs. S2–S7 in Supporting Information). On another extreme, 12% (3/25) of plant-seed dispersal networks show a slight decrease in *Q*_*e*_ as the rewiring probability is increased, while for the rest it remains rather constant (12%, 12/101, for plant-pollinator networks and 28%, 7/25, for plant-seed dispersal networks). We clustered the empirical mutualistic networks and constructed a dendrogram of the empirical mutualistic networks based on the similarities of the response of the effective modularity to extinctions and rewiring (see Fig. S8 in Supporting Information). In general, most networks show an increment in *Q*_*e*_ with rewiring. The directed extinction together with resource affinity rewiring (*i*.*e*., the DF scenario) increases *Q*_*e*_ more than any of the other scenarios. In the bottom panel of Fig. 4, the difference between RR and DF scenarios is shown.

In order to assess the average persistence of the network, we computed the averages over all node pairs to get the mean persistence *P*_*M*_ = 〈Π_*ij*_(*g*_*i*_ = *h*_*j*_, *t*|*g*_*i*_ = *h*_*j*_, *t*_0_)〉. The extinction mechanism at play affects the results the most, as was the case for the robustness. The persistence of communities for random extinctions decays almost in a linear fashion as a function of the fraction of extinct resources, with a 50% probability to remain in the same community after 40% of the resources are removed. In contrast, for the directed extinction scenario, persistence decays to a baseline value of less than 30% when less than 20% of resources are removed (values correspond to the coral reef network but results for the rest of networks are qualitatively similar, leading to a higher reconfiguration into new communities with improved compartmentalization. The rewiring scenario increases the persistence of the community structure when the rewiring is driven by resource affinity, which implies that functional replacement helps module persistence. To quantify the gain in persistence due to resource affinity-driven rewiring, we compared the probability that two random nodes (resource or consumer) are in the same module for each extinction scenario but different adaptation, that is, we compared, on the one hand, RR with RF scenarios, and on the other, DR with DF. In both cases, resource affinity-driven rewiring leads to higher mean persistence values, (see Figs. S14 and S15 in Supporting Information).

### Stability

**Figure 5:**
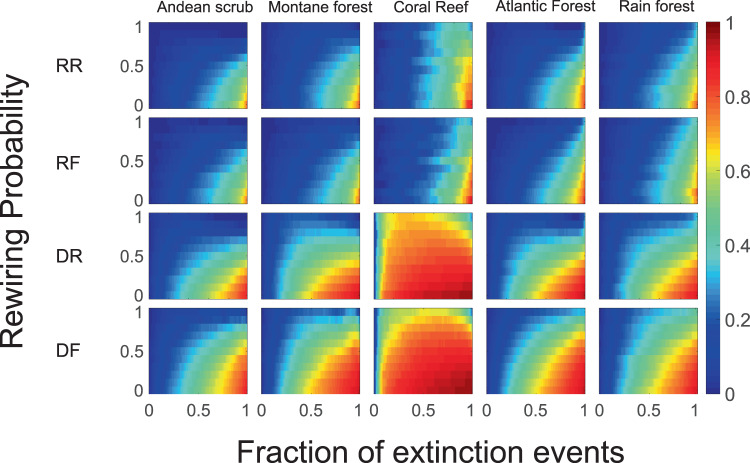
Stability of mutualistic networks for the different extinction-adaptation scenarios. Each column shows the stability (in a color scale from blue, 〈Σ〉 = 0, to red, 〈Σ〉 = 1) for the five bipartite networks (Table 1) as a function of the probability of rewiring and the fraction of extinct nodes. Each row corresponds to a different extinction-adaptation scenario. Results are averaged over 100 realizations of the extinction-adaptation sequences for the different scenarios.

Similarly to robustness and community persistence, stability is affected more heavily by the extinction rule than by the adaptation rule (Fig. 5). Regardless of the adaptation rule, robustness is conserved, as indicated by the relative change of the leading eigenvalue remaining close to zero as long as the network does not break up in components for large values of the fraction of extinct nodes.

The stability of the bipartite networks increases when a high percentage of species are removed, that is, once the original network is reduced to a few species. For directed extinctions, and also irrespective of the adaptation rule, stability increases much more than for random extinctions, with the effect being more noticeable for a lower fraction of extinction-adaptation events. In these cases, the effect also decreases as the rewiring probability *r* increases. We also note that in targeted extinction scenarios, the average connectivity of the network decreases with fewer extinctions, as we are taking away individuals with large degrees, and thus stability increases. The results for the rest of the networks are qualitatively similar (see Figs. S9–S11 in Supporting Information).

### Effective modularity and the normalized maximum degree *k*_max_

**Figure 6:**
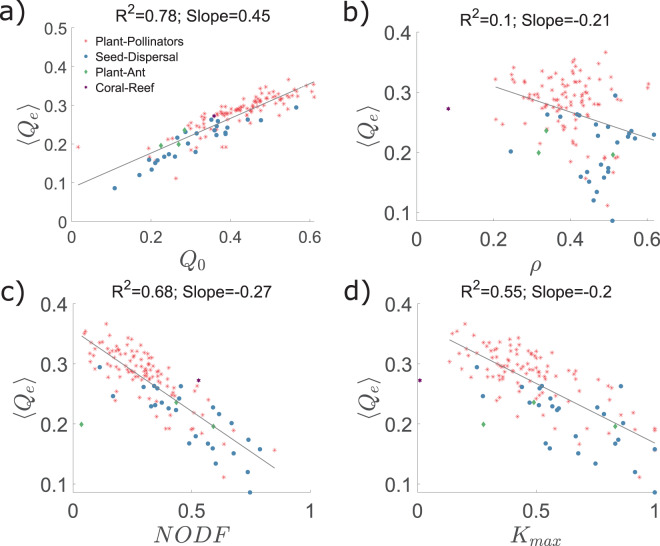
Correlation of the average effective modularity with the original modularity, nestedness, the normalized largest degree, and robustness. The average effective modularity is calculated over all extinction fractions and rewiring values in $p\in [0,1]$. It correlates positively with (**a**) the modularity of the original network and (**d**) the robustness averaged for all rewiring parameters. The correlation is negative with (**b**) nestedness (measured as NODF^60^) and with (**c**) the normalized largest degree of the original network. Values are obtained for directed extinction and resource affinity adaptation. PP (red asterisks): plant-pollinator networks; SD (blue circles): seed dispersal networks; PA (green diamonds): plant-ant networks; Coral Reef (purple stars).

In order to know if the transformation of the modular structure of bipartite networks over time can be anticipated by properties of the original network, we computed the average effective modularity 〈*Q*_*e*_〉 of the networks for all rewiring probabilities and all fractions of extinction events (average of the plots in Fig. 4 and correlated it with different initial topological characteristics (see Fig. 6). As *Q*_*e*_ behaves very similarly for all scenarios, we only used the DF scenario. The average *Q*_*e*_ is positively correlated to the modularity of the original network *Q*_0_ (*R*^2^ = 0.78, Fig. 6a) but uncorrelated to its robustness when this is measured for random extinctions and no rewiring (*R*^2^ = 0.1, Fig. 6b). On the other hand, *Q*_*e*_ correlates negatively with nestedness, measured as NODF^60^ (*R*^2^ = 0.68, Fig. 6c) and with the normalized largest degree *K*_*max*_, which is the largest degree in the network divided by max(*n*, *m*) (*R*^2^ = 0.55, Fig. 6d). All these results can thus be reframed in terms of the normalized largest degree, *K*_*max*_: the larger *K*_*max*_ of the original network, the lower the effective modularity and robustness, and the larger the nestedness.

## Discussion

Mutualistic consumer-resource species interactions in ecosystems are well described by bipartite networks, where links inside the two groups of species are not allowed (between consumers and between resources)^61–64^. We explored the effects of local species extinction followed by different strategies to adaptation on the robustness, modularity, and stability of 130 empirical mutualistic bipartite ecological networks^53–55^ under the assumptions that i) no new species enter the network and ii) only the binary presence/absence of interactions is considered (not interaction strength). For heterogeneous weight distributions, the results might differ and the dynamical rules have to be formulated to account for the weights. A parsimonious plasticity rule will introduce the rewiring probability as proportional to the weights, thus favoring species with interactions with the largest weights. This will likely introduce rich dynamics and can affect network plasticity depending on whether specialists or generalists have the strongest interactions.

Altogether, we explored four extinction-adaptation scenarios to conclude that the extinction mechanism is crucial to determine the fate of the system concerning its robustness and stability. Directed extinctions lead to new networks that are less robust and more stable, an effect that decreases with growing rewiring probability. This result goes in the same direction as the seminal work by May^44^: more complex and highly interconnected ecosystems are less likely to be stable. The trend we observe is that resource affinity rewiring leads to an increase of modularity, stability, and robustness in comparison to a random rewiring, which is used as a parsimonious rewiring. All these three properties are desirable for a mutualistic network as it provides resilience to disturbances. Thus resource affinity could be selected as an evolutionary mechanism to increase the survival of ecosystems. Earlier studies suggest that the adaptation mechanism in the topological approach increases the robustness of mutualistic networks to species removal^33,35,65–67^. Our results show that topological plasticity increases robustness, in agreement with those studies. Our simulations also suggest that resource affinity consumer rewiring induced by preferred species extinction is more detrimental to the robustness of the network than random rewiring.

The increase in robustness is connected to changes in other structural metrics: modularity, stability, and robustness are positively correlated among them, but all negatively correlated with nestedness. Our results are consistent with the trends reported in these metrics although the literature shows some conflicting outcomes. Modularity and nestedness are observed to be negatively correlated, as found in several bipartite networks^68^, at least for large connectance^43^ and especially for pollination networks^69^. Nevertheless, note that this relation is not mathematically proven except for the case of some models as in^68^. Stability is negatively correlated with nestedness as reported in some studies^70,71^, but positively correlated with modularity, in agreement with previous studies^14,72,73^. Positive correlations between robustness and modularity have been reported recently in plant-frugivore networks^74^. However, our results show a non-significant positive correlation between robustness and modularity.

The assumption that resource affinity may affect rewiring is based on the hypothesis that co-occurring species are functionally linked or equivalent. However, resource affinity rewiring patterns are rarely studied in ecosystems. In biological terms, such rewiring might imply that the likelihood that a consumer would use a particular resource used by a different species increases with the number of resources it shares with that species. In real ecosystems, there are several rewiring strategies taking place at the same time^75^. There is evidence for rewiring based on abundance^76–78^, temporal overlap^75,79^, trait matching^79–83^, and trait resemblance^84,85^. Temporal overlap, based on the phenology of the species, is a necessary condition for a rewiring among species to take place. So is it, to a certain degree, the abundance of species. Given that, species interactions might be constrained by the physiological capacity of one to interact with another, which is described by trait matching. This is the case for example in birds that can only pollinate certain species of flowers if the length of their bills coincides with the corolla length of the flowers. Another different but related case happens when a consumer decides to feed on a resource because another consumer species has a similar trait as his own. This is described by trait resemblance. Resource-affinity rewiring is a proxy for both trait matching and trait resemblance. On the one hand, in the absence of information about traits we expect that trait matching increases with resource overlap, that is, the more common resources are shared between pair of species more likely is that their traits match, On the other hand, we are using a stochastic approach, thus species can rewire to a resource that does not necessarily have the largest overlap, we are taking into account also trait resemblance. Trait matching is stricter than trait resemblance, and thus future innovations will require to consider nonlinear functions of the overlap to capture trait matching more precisely (for example with a threshold value for rewiring). The rewiring based on consumer overlap has been used in some literature, measures as Sorensen similarity index between a pair of plants proportional to the number of sharing pollinators over the sum of the pollinator species of both plants. This approach assumes that topological similarity is associated with phenotypic similarity (trait resemblance)^86^.

Through analyzing a broad spectrum of bipartite mutualistic networks, we make the argument that evolutionary responses to the loss of species lead networks to become more modular, and the effect is largest when well-connected species were removed from the networks (directed extinctions). These responses could act in the same generation, as a behavioral response, or between generations as an evolutionary mechanism. Our results further demonstrate that adaptation in the form of resource affinity-driven consumer rewiring has the most significant impact on the degree of compartmentalization of the networks. This modular configuration of the network increases the capacity of absorbing disturbance (for example species loss) and hence has a positive effect on the dynamical stability of mutualistic networks. The effective network modularity shows a similar pattern for any of the extinction scenarios but depends on details of the original bipartite network, that is, the effective modularity is idiosyncratic. We also observed a marginal increase in effective modularity with resource affinity driven rewiring. Experiments show that, in agreement with theory, networks with a modular structure of species interactions benefit from localized dispersion of extinction^87^. Whether this is the case in nature is not resolved, as dynamic rewiring of bipartite mutualistic networks following local extinctions are still poorly documented.

Different scenarios lead a different number of unsuccessful rewiring attempts, thus some scenarios lose more links per extinction event than others. This can happen in two ways, either because the proposed rewired link already exists or because there are no plausible partners for the rewiring. The former happens for both rewirings, while the latter only in the resource-affinity rewiring. Nevertheless, we speculate that this consequence of resource-affinity rewiring can be interpreted as the existence of forbidden links^88^. Although there are many available resource species in the network, the set of plausible resources to rewire to is empty, as no other consumer that also used the extinct resource uses any of the available resources.

We have shown that for the empirical bipartite networks, the normalized largest degree is correlated with the modularity of the original network. Thus, the success of niche compartmentalization with rewiring probability can be anticipated with the (normalized) largest degree. At the same time, nestedness showed to decrease as modularity increases, nestedness decreases. Finally, stability decreases as the largest degree correlate with stability. How ecosystems face disruptive, extreme events or continuous environmental changes cannot be predicted generically and requires specific knowledge on the species that will be most likely affected and the plasticity of the remaining species to reorganize the whole network. Our experiments with bipartite mutualistic networks consistently show that functional replacement, here modeled as resource affinity, can enhance the resilience of mutualistic networks by increasing the effective modularity as the bipartite networks lose species. Resource affinity is just a proxy for functional redundancy, which has been argued to add resilience to ecosystems under disturbance^89,90^. The role of functional diversity in ecosystem stability has been previously explored based on simulated random extinctions^84^, but directed extinctions, as simulated here, were not assessed, nor was resource affinity used as a proxy of functional redundancy in previous simulation studies. Our analysis of the resilience of bipartite mutualistic networks predicts that functional redundancy should help stabilize biological networks against local extinctions. We put forward the hypothesis that resource affinity, as a proxy for functional redundancy, is a likely rewiring mechanism of mutualistic networks in the presence of local extinctions. That is to say, ecosystems have a higher chance of reconfiguration under species extinction. Testing these predictions through longitudinal observations of bipartite mutualistic networks or direct experimental manipulation is required to assess the value of resource affinity for ecosystems resilience, and implementing data-based plasticity mechanisms.

## Supplementary information

Supplementary Information.

## Data Availability

We collected 130 empirical mutualistic networks from the literature and databases (see Supporting Information Table S1). In particular, 129 networks were collected from Web of Life: ecological networks database (available in http://www.web-of-life.es), and one Host-Symbiodiniaceae network was obtained from coral reefs^54,55^.
