# Author Correction: Robustness to extinction and plasticity derived from mutualistic bipartite ecological networks

**DOI:** 10.1038/s41598-020-72922-7

**Published:** 2020-09-29

**Authors:** Somaye Sheykhali, Juan Fernández-Gracia, Anna Traveset, Maren Ziegler, Christian R. Voolstra, Carlos M. Duarte, Víctor M. Eguíluz

**Affiliations:** 1 Instituto de Física Interdisciplinar Y Sistemas Complejos IFISC (CSIC-UIB), 07122 Palma de Mallorca, Spain; 2 Instituto Mediterráneo de Estudios Avanzados IMEDEA (CSIC-UIB), 07121 Esporles, Spain; 3 Department of Animal Ecology and Systematics, Justus Liebig University, Heinrich-Buf-Ring 26-32 IFZ, 35392 Giessen, Germany; 4 Department of Biology, University of Konstanz, 78457 Konstanz, Germany; 5 Red Sea Research Center, King Abdullah University of Science and Technology, Thuwal, Kingdom of Saudi Arabia

Correction to: *Scientific Reports* https://doi.org/10.1038/s41598-020-66131-5, published online: 17 June 2020.

The original Supplementary Information file published with this Article was incorrect. This version did not include Figure S1, Figure S2, Figure S3, Figure S4, Figure S5, Figure S6, Figure S7, Figure S8, Figure S9, Figure S10, Figure S11, Figure S12, Figure S13, Figure S14 or Figure S15. The correct Supplementary Information file now accompanies the Article.

