# Supplementary material for: Robustness to extinction and plasticity derived from mutualistic bipartite ecological networks
Source: Sci Rep. 2020 Jun 17;10:9783. doi: 10.1038/s41598-020-66131-5 (PMC7300072; doi:10.1038/s41598-020-66131-5)
Supplement: Supplementary file 1 — Supplementary Information. [file 41598_2020_66131_MOESM1_ESM.pdf]

SUPPLEMENTARY INFORMATION FOR:
Robustness to extinction and plasticity derived from mutualistic bipartite ecological networks

Somaye Sheykhali[1], Juan Fernández-Gracia[1,*], Anna Traveset[2], Maren Ziegler[3], Christian R. Voolstra[4], Carlos M. Duarte[5], and Víctor M. Eguíluz[1]

[1]Instituto de Física Interdisciplinar y Sistemas Complejos IFISC (CSIC - UIB), Palma de Mallorca, E-07122, Spain
[2]Instituto Mediterráneo de Estudios Avanzados IMEDEA (CSIC-UIB), E07121 Esporles, Spain
[3]Department of Animal Ecology & Systematics, Justus Liebig University, Heinrich-Buff-Ring 26-32 IFZ, 35392 Giessen, Germany
[4]Department of Biology, University of Konstanz, Konstanz 78457, Germany
[5]Red Sea Research Center, King Abdullah University of Science and Technology, Thuwal, Kingdom of Saudi Arabia
[*]juanf@ifisc.uib-csic.es

ABSTRACT

In this supplementary information we provide a table describing all of the networks in the study and figures showing the results of the analysis for all the networks that are not shown in the main text. The database[1] includes many webs with a wide range of network sizes, but here we only use those in which the network size is big enough to study the extinction events sequences (≥ 7). Specifically, we used 130 interaction networks involving 101 plant-pollinator, 25 seed-dispersal, 3 plant-ant, and 1 host-symbiont association (see table S1 for the description of each dataset).

Contents

S1 Elements about the data set 2
S2 Robustness for different extinction and adaptive rewiring scenarios 5
S3 Effective modularity for Random extinction – Random adaptive rewiring scenario 6
S4 Effective modularity for Random extinction – Resource-Affinity adaptive rewiring scenario 7
S5 Effective modularity for Directed extinction – Random adaptive rewiring scenario 8
S6 Effective modularity for Directed extinction – Resource-Affinity adaptive rewiring scenario 9
S7 The difference in the effective modularity between DR and DF scenarios 10
S8 The difference in the effective modularity between RF and RR scenarios 11
S9 Similarities of the response of the effective modularity to extinctions and adaptive rewiring 12
S10 Stability for Random extinction – Random adaptive rewiring scenario 13
S11 Stability for Directed extinction – Random adaptive rewiring scenario 14
S12 Stability for Directed extinction – Resource-Affinity adaptive rewiring scenario 15
S13 The difference between DR-DF scenarios 16
S14 The difference in the stability between RR-RF scenarios 17
S15 The module persistence for Directed extinction – Resource-Affinity adaptive rewiring scenario 18
S16 The difference in the community distribution between RR-DF scenarios 19
References 20

S1 Elements about the data set

Table S1. Ecological webs of mutualistic interactions

Habitat type	Species interactions		Interactions
Andean scrub, Chile[2]	43–Plants	62–Pollinators	361
Andean scrub, Chile[2]	87–Plants	98–Pollinators	196
Andean scrub, Chile[2]	41–Plants	28–Pollinators	81
Boreal forest[3]	12–Plants	102–Pollinators	167
CaatingaCaatinga[4]	96–Plants	276–Pollinators	923
Montane forest, UK[5]	17–Plants	61–Pollinators	146
Montane forest, UK[5]	16–Plants	36–Pollinators	85
Coastal forest, Canary Islands[6]	11–Plants	38–Pollinators	106
Alpine subarctic, Sweden[7]	24–Plants	118–Pollinators	242
Alpine subarctic, Sweden[7]	31–Plants	76–Pollinators	456
Coastal forest, Mauritius Island[8]	14–Plants	13–Pollinators	52
Rocky cliff and open herb community[8]	10–Plants	12–Pollinators	30
Coastal forest, South Africa[9]	9–Plants	56–Pollinators	103
Arctic community, Canada[10]	29–Plants	86–Pollinators	179
Arctic community, Spain[11]	26–Plants	179–Pollinators	412
Montane forest, England[12]	25–Plants	79–Pollinators	299
Medow, Australia[13]	42–Plants	91–Pollinators	264
High Arctic, Canada[14]	32–Plants	115–Pollinators	190
Beech forest, Kyoto, Japan[15]	93–Plants	679–Pollinators	1193
Xeric scrub, Argentina[16]	21–Plants	45–Pollinators	83
Arctic community, Canada[17]	11–Plants	18–Pollinators	38
Deciduous forest, USA[18]	13–Plants	44–Pollinators	143
Galapagos, Galapagos[19]	106–Plants	54–Pollinators	204
New Zealand, New Zealand[20]	18–Plants	60–Pollinators	120
Mountain flora, New Zealand[20]	41–Plants	139–Pollinators	374
Mountain flora, New Zealand[20]	49–Plants	118–Pollinators	346
Palm swamp community, Venezuela[21]	33–Plants	53–Pollinators	109
Palm swamp community, Venezuela[22]	48–Plants	49–Pollinators	156
Maple-oak woodland, USA[23]	7–Plants	32–Pollinators	65
Peat bog, Canada[24]	13–Plants	34–Pollinators	141
Rain forests, Chile[25]	26–Plants	128–Pollinators	312
Peat bog, Jamaica[26]	61–Plants	36–Pollinators	178
Hestehaven, Denmark[27]	10–Plants	40–Pollinators	72
Hestehaven, Denmark[8]	144–Plants	Pollinators	383
Hestehaven, Denmark[27]	110–Plants	Pollinators	250
Puerto Villamil, Galapagos[28]	12–Plants	6–Pollinators	25
Greenland[29]	17–Plants	26–Pollinators	63
Denmark[30]	16–Plants	44–Pollinators	278
Greenland[29]	17–Plants	26–Pollinators	63
Heathland, Isenbjerg[31]	19–Plants	186–Pollinators	425
heathland[31]	30–Plants	236–Pollinators	671
Danish forest meadow, Denmark[32]	37–Plants	225–Pollinators	262
Canary Islands[33]	14–Plants	35–Pollinators	86
Tundra, Greenland[34]	15–Plants	39–Pollinators	92
Mediterranean shrub lands[35]	32–Plants	81–Pollinators	319
Caatinga (semi-arid vegetation)[36]	13–Plants	13–Pollinators	71
Reserva Florestal Brazil[37]	14–Plants	8–Pollinators	32
Estacion de Biologia Chamela, Jalisco[38]	21–Plants	5–Pollinators	44

Continued on next page

Table S1-*Continued from previous page*

Habitat type	Species interactions		Interactions
Woody riverine vegetation and xeric scrub, Argentina[16]	23–Plants	72–Pollinators	125
Tenerife, Canary Islands[33]	17–Plants	51–Pollinators	129
Windsor, The Cockpit Country, Jamaica[39]	31–Plants	43–Pollinators	114
Syndicate, Dominica[39]	17–Plants	26–Pollinators	145
Evergreen montane forest, Argentina[40]	44–Plants	8–Pollinators	164
Heathland habitat, Mauritius[41]	12–Plants	38–Pollinators	107
Heathland habitat, Mauritius[41]	13–Plants	45–Pollinators	196
Heathland habitat, Mauritius[41]	21–Plants	46–Pollinators	196
Heathland habitat, Mauritius[41]	33–Plants	54–Pollinators	196
Heathland habitat, Mauritius[41]	26–Plants	45–Pollinators	96
Heathland habitat, Mauritius[41]	29–Plants	39–Pollinators	108
Heathland habitat, Mauritius[41]	19–Plants	28–Pollinators	72
Heathland habitat, Mauritius[41]	18–Plants	40–Pollinators	78
Heathland habitat, Mauritius[41]	14–Plants	25–Pollinators	51
Heathland habitat, Mauritius[41]	14–Plants	20–Pollinators	43
Heathland habitat, Mauritius[41]	11–Plants	26–Pollinators	60
Heathland habitat, Mauritius[41]	7–Plants	31–Pollinators	48
Heathland habitat, Mauritius[41]	11–Plants	37–Pollinators	99
Heathland habitat, Mauritius[41]	14–Plants	37–Pollinators	131
Heathland habitat, Mauritius[41]	17–Plants	39–Pollinators	114
Heathland habitat, Mauritius[41]	17–Plants	35–Pollinators	100
Heathland habitat, Mauritius[41]	20–Plants	28–Pollinators	75
Heathland habitat, Mauritius[41]	18–Plants	13–Pollinators	41
Heathland habitat, Mauritius[41]	12–Plants	18–Pollinators	37
Heathland habitat, Mauritius[41]	13–Plants	31–Pollinators	64
Heathland habitat, Mauritius[41]	12–Plants	27–Pollinators	57
Heathland habitat, Mauritius[41]	14–Plants	24–Pollinators	46
Heathland habitat, Mauritius[42]	8–Plants	14–Pollinators	29
Heathland habitat, Mauritius[42]	12–Plants	22–Pollinators	51
Heathland habitat, Mauritius[42]	11–Plants	24–Pollinators	58
Heathland habitat, Mauritius[42]	9–Plants	23–Pollinators	62
Heathland habitat, Mauritius[42]	7–Plants	12–Pollinators	24
Heathland habitat, Mauritius[42]	7–Plants	19–Pollinators	39
Heathland habitat, Mauritius[42]	8–Plants	19–Pollinators	41
Heathland habitat, Mauritius[42]	9–Plants	19–Pollinators	41
Heathland habitat, Mauritius[42]	11–Plants	35–Pollinators	41
Heathland habitat, Mauritius[42]	11–Plants	44–Pollinators	41
Heathland habitat, Mauritius[42]	11–Plants	25–Pollinators	41
Heathland habitat, Mauritius[42]	11–Plants	43–Pollinators	41
Heathland habitat, Mauritius[42]	10–Plants	18–Pollinators	28
Heathland habitat, Mauritius[42]	10–Plants	26–Pollinators	45
Heathland habitat, Mauritius[42]	11–Plants	18–Pollinators	46
Heathland habitat, Mauritius[42]	7–Plants	13–Pollinators	28
Heathland habitat, Mauritius[42]	8–Plants	19–Pollinators	36
Heathland habitat, Mauritius[42]	8–Plants	18–Pollinators	39
Heathland habitat, Mauritius[42]	7–Plants	13–Pollinators	30
Heathland habitat, Mauritius[42]	9–Plants	13–Pollinators	19
Heathland habitat, Mauritius[42]	7–Plants	17–Pollinators	31
Heathland habitat, Mauritius[42]	11–Plants	19–Pollinators	43
Heathland habitat, Mauritius[42]	10–Plants	23–Pollinators	49
Heathland habitat, Mauritius[42]	7–Plants	23–Pollinators	46
Heathland habitat, Mauritius[42]	9–Plants	26–Pollinators	58

Continued on next page

Table S1-*Continued from previous page*

**Habitat type**	**Species interactions**		**Interactions**
Heathland habitat, Mauritius[42]	9–Plants	16–Pollinators	26
Heathland habitat, Mauritius[42]	10–Plants	11–Pollinators	23
Heathland habitat, Mauritius[42]	11–Plants	23–Pollinators	50
Heathland habitat, Mauritius[42]	13–Plants	21–Pollinators	45
Heathland habitat, Mauritius[42]	9–Plants	26–Pollinators	57
Heathland habitat, Mauritius[42]	9–Plants	14–Pollinators	32
Santa Virginia Field Station[43]	56–Plants	10–Pollinators	123
Rainforest, Colombia[44]	44–Plants	8–Pollinators	89
Princeton, USA[45]	7–Plants	21–seed dispersers	50
Mount Missim, New Guinea[46]	31–Plants	9–seed dispersers	119
Caguana, Puerto Rico[47]	25–Plants	16–seed dispersers	68
Cialitos, Puerto Rico[47]	34–Plants	20–seed dispersers	95
Cordillera, Puerto Rico[47]	25–Plants	13–seed dispersers	49
Fronton, Puerto Rico[47]	21–Plants	15–seed dispersers	51
Tropical rain-forest, Australia[48]	72–Plants	7–seed dispersers	143
Mtunzini, South Africa[49]	16–Plants	10–seed dispersers	110
Santa Genebra Reserve[50]	7–Plants	18–seed dispersers	38
Tropical rainforest, Trinidad.[51]	50–Plants	14–seed dispersers	234
Calton, Great Britain[52]	11–Plants	14–seed dispersers	47
Santa Genebra Reserve, Brazil[50]	35–Plants	29–seed dispersers	146
North Negros Forest Reserve, Philippine[53]	36–Plants	19–seed dispersers	197
Hato Raton, Sevilla, Spain[54]	16–Plants	17–seed dispersers	121
Kuala Lompat, Reserve[55]	24–Plants	61–seed dispersers	500
Gabon, Africa[56]	16–Plants	8–seed dispersers	72
Kuala Lompat, Reserve[57]	24–Plants	32–seed dispersers	66
Monteverde, Costa Rica[58]	196–Plants	40–seed dispersers	666
Intervales and Saibadela, Brazil[59]	207–Plants	110–seed dispersers	1121
Yakushima Island, Japan[60]	15–Plants	8–seed dispersers	38
NW Spain temperate forest[61]	12–Plants	7–seed dispersers	40
Serra da Tronqueira[62]	42–Plants	8–seed dispersers	84
Soberania National Park[63]	13–Plants	11–seed dispersers	53
Estacion Biologica de Cocha Cashu[64]	16–Plants	8–Ants	19
Forest Fragments[65]	24–Plants	15–Ants	45
Australian Canopy Crane[66]	41–Plants	48–Ants	284

S2 Robustness for different extinction and adaptive rewiring scenarios

S1-eps-converted-to.pdf

Figure S1. Robustness for the extinction - adaptive rewiring scenarios. In each panel the x-axis is the rewiring probabilities, $r \in [0,1]$, and the y-axis the robustness, ρ, averaged over 100 realizations of the extinction-rewiring sequences for the different scenarios. Each block displays the results for each data sets: plant-pollinators, plant-seed dispersers and plant-ants; Results are averaged over 100 realizations of the extinction-rewiring sequences for the different scenarios.

S3 Effective modularity for Random extinction – Random adaptive rewiring scenario

S2-eps-converted-to.pdf

Figure S2. Effective modularity for the Random extinction – Random adaptive rewiring scenario. The modularity is represented in a color scale from $Q_e = 0$ (blue) to $Q_e = 0.5$ (red), in each panel the x-axis is the fraction of extinction events, and the y-axis the rewiring probability r. Each block displays the results for each data sets: plant-pollinators, plant-seed dispersers and plant-ants; reported values are averaged over 100 realizations of the extinction-rewiring sequences for the different scenarios.

S4 Effective modularity for Random extinction – Resource-Affinity adaptive rewiring scenario

S3-eps-converted-to.pdf

Figure S3. Effective modularity for the Random extinction – Resource-Affinity adaptive rewiring scenario. The modularity is represented in a color scale from $Q_e = 0$ (blue) to $Q_e = 0.5$ (red), in each panel the x-axis is the fraction of extinction events, and the y-axis the rewiring probability r. Each block displays the results for each data sets: plant-pollinators, plant-seed dispersers and plant-ants; reported values are averaged over 100 realizations of the extinction-rewiring sequences.

S5 Effective modularity for Directed extinction – Random adaptive rewiring scenario

S4-eps-converted-to.pdf

Figure S4. Effective modularity for the Directed extinction – Random adaptive rewiring scenario. The effective modularity is represented in a color scale from $Q_e = 0$ (blue) to $Q_e = 0.5$ (red), in each panel the x-axis is the fraction of extinction events, and the y-axis the rewiring probability r. Each block displays the results for each data sets: plant-pollinators, plant-seed dispersers and plant-ants; reported values are averaged over 100 realizations of the extinction-rewiring sequences.

S6 Effective modularity for Directed extinction – Resource-Affinity adaptive rewiring scenario

S5-eps-converted-to.pdf

Figure S5. Effective modularity for the Directed extinction – Resource-Affinity adaptive rewiring scenario. The effective modularity is represented in a color scale from $Q_e = 0$ (blue) to $Q_e = 0.5$ (red), in each panel the x-axis is the fraction of extinction events, and the y-axis the rewiring probability r. Each block displays the results for each data sets: plant-pollinators, plant-seed dispersers and plant-ants; Results are averaged over 100 realizations of the extinction-rewiring sequences.

S7 The difference in the effective modularity between DR and DF scenarios

S6-eps-converted-to.pdf

Figure S6. The difference in the effective modularity for the same ecosystem and reported values are averaged over 100 realizations of the Directed extinction – Random rewiring sequences and Directed extinction-Resource-Affinity adaptive rewiring.

S8 The difference in the effective modularity between RF and RR scenarios

S7-eps-converted-to.pdf

Figure S7. The difference in the effective modularity for the same ecosystem and the results are averaged over 100 realizations of the Random extinction – Resource-Affinity rewiring sequences and Random extinction-Random rewiring.

S9 Similarities of the response of the effective modularity to extinctions and adaptive rewiring

S8-eps-converted-to.pdf

Figure S8. Dendrogram of the empirical mutualistic networks based on the similarities in the response of the effective modularity to extinctions and adaptive rewiring

S10 Stability for Random extinction – Random adaptive rewiring scenario

S9-eps-converted-to.pdf

Figure S9. Stability for Random extinction – Random adaptive rewiring scenario. Each panel illustrates the stability of one data set (Table S1) as a function of the probability of rewiring and fraction of extinction events. Results are averages over 100 different realizations. Colors scaled from red, maximum stability to blue, minimum stability.

S11 Stability for Directed extinction – Random adaptive rewiring scenario

S10-eps-converted-to.pdf

Figure S10. Stability for Directed extinction – Random adaptive rewiring scenario. Each panel illustrates the stability of one data set (Table S1) as a function of the probability of rewiring and fraction of extinction events. Results are averages over 100 different realizations. Colors scaled from red, maximum stability to blue, minimum stability.

S12 Stability for Directed extinction – Resource-Affinity adaptive rewiring scenario

S11-eps-converted-to.pdf

Figure S11. Stability for Directed extinction – Resource-Affinity adaptive rewiring scenario. Each panel illustrates the stability of one data set (Table S1) as a function of the probability of rewiring and fraction of extinction events. Results are averages over 100 different realizations. Colors scaled from red, maximum stability to blue, minimum stability.

S13 The difference between DR-DF scenarios

S12-eps-converted-to.pdf

Figure S12. The difference between stability in the presence of Directed extinction – Resource-Affinity rewiring scenario and Random extinction –Random adaptive rewiring scenario of one data set as a function of the probability of rewiring and fraction of extinction events.

S14 The difference in the stability between RR-RF scenarios

S13-eps-converted-to.pdf

Figure S13. The difference between stability in the presence of Directed extinction – Resource-Affinity adaptive rewiring scenario and Random extinction – Random adaptive rewiring scenario of one data set as a function of the probability of adaptive rewiring and fraction of extinction events.

S15 The module persistence for Directed extinction – Resource-Affinity adaptive rewiring scenario

The module persistence Π_{ij} defined as the probability that two nodes remain in the same community if they were initially in the same community, $\Pi(\mu_i = \mu_j, t | \mu_i = \mu_j, t_0)$, where μ_i (μ_j) is the community to which node i (j) belongs.

S14-eps-converted-to.pdf

Figure S14. The module persistence for Directed extinction – resource-affinity adaptive rewiring scenario (DF) in the Coral Reef data set.

S16 The difference in the community distribution between RR-DF scenarios

S15-eps-converted-to.pdf

Figure S15. The difference in the community distribution between RR-DF scenarios

References

1. Fortuna, M. A., Ortega, R. & Bascompte, J. The web of life. Available at www.web-of-life.es (Date accessed: 2018).
2. Arroyo, M. T. K., Primack, R. & Armesto, J. Community studies in pollination ecology in the high temperate Andes of central Chile. I. Pollination mechanisms and altitudinal variation. *Am. J. Bot.* **69**, 82–97 (1982).
3. Barrett, S. C. & Helenurm, K. The reproductive biology of boreal forest herbs. i. breeding systems and pollination. *Can. J. Bot.* **65**, 2036–2046 (1987).
4. Clements, F. E. & Long, F. L. *Experimental pollination: an outline of the ecology of flowers and insects*, vol. 336 (Carnegie Institute of Washington, Washington, D.C., USA, 1923).
5. Dicks, L. V., Corbet, S. A. & Pywell, R. F. Compartmentalization in plant-insect flower visitor webs. *J. Animal Ecol.* **71**, 32–43 (2002).
6. Dupont, Y. L., Hansen, D. M. & Olesen, J. M. Structure of a plant-flower-visitor network in the high-altitude sub-alpine desert of Tenerife, Canary Islands. *Ecography* **26**, 301–310 (2003).
7. Elberling, H. & Olesen, J. M. The structure of a high latitude plant-flower visitor system: the dominance of flies. *Ecography* **22**, 314–323 (1999).
8. Olesen, J. M., Eskildsen, L. I. & Venkatasamy, S. Invasion of pollination networks on oceanic islands: importance of invader complexes and endemic super generalists. *Divers. Distributions* **8**, 181–192 (2002).
9. Ollerton, J., Johnson, S. D., Cranmer, L. & Kellie, S. A. M. The pollination ecology of an assemblage of grassland asclepiads in South Africa. *Annals Bot.* **92**, 807–834 (2003).
10. Hocking, B. Insect-flower associations in the high Arctic with special reference to nectar. *Oikos* **19**, 359–387 (1968).
11. Herrera, J. Pollination relationships in southern Spanish Mediterranean shrublands. *The J. Ecol.* **76**, 274–287 (1988).
12. Memmott, J. The structure of a plant-pollinator food web. *Ecol. Lett.* **2**, 276–280 (1999).
13. Inouye, D. W. & Pyke, G. H. Pollination biology in the Snowy Mountains of Australia: comparisons with montane Colorado, USA. *Aust. J. Ecol.* **13**, 191–205 (1988).
14. Kevan, P. G. *High Arctic Insect-flower Relations: The Inter-relationships of Arthropods and Flowers at Lake Hazen* (Department of Entomology, University of Alberta, Ellesmere Island, N.W.T., Canada, 1970).
15. Kato, M., Kakutani, T., Inoue, T. & Itino, T. Insect-flower relationship in the primary beech forest of Ashu, Kyoto: an overview of the flowering phenology and the seasonal pattern of insect visits. *Contributions from Biol. Lab. Kyoto Univ.* **27**, 377–463 (1990).
16. Medan, D. *et al.* Plant-pollinator relationships at two altitudes in the Andes of Mendoza, Argentina. *Arctic, Antarctic, Alp. Res.* **34**, 233 (2002).
17. Mosquin, T. Observations on the pollination biology of plants on Melville Island, NWT. *Can. Fld Nat* **81**, 201–205 (1967).
18. Motten, A. F., Campbell, D. R., Alexander, D. E. & Miller, H. L. Pollination effectiveness of specialist and generalist visitors to a North Carolina population of Claytonia virginica. *Ecology* **62**, 1278–1287 (1981).
19. McMullen, C. K. Flower-visiting insects of the Galapagos Islands. *Pan-Pacific Entomol.* **69**, 95–106 (1993).
20. Primack, R. B. Insect pollination in the New Zealand mountain flora. *New Zealand J. Bot.* **21**, 317–333 (1983).
21. Ramirez, N. & Brito, Y. Pollination biology in a palm swamp community in the Venezuelan Central Plains. *Bot. J. Linnean Soc.* **110**, 277–302 (1992).
22. Ramirez, N. Biología de polinización en una comunidad arbustiva tropical de la alta Guayana venezolana. *Biotropica* **21**, 319–330 (1989).
23. Schemske, D. W. *et al.* Flowering ecology of some spring woodland herbs. *Ecology* **59**, 351–366 (1978).
24. Small, E. Insect pollinators of the Mer Bleue peat bog of Ottawa. *Can. field-naturalist* **2**, 351–366 (1976).
25. Smith-Ramírez, C., Martinez, P., Nunez, M., González, C. & Armesto, J. J. flower visitation frequency and generalism of pollinators in temperate rain forests of Chiloé Island, Chile. *Bot. J. Linnean Soc.* **147**, 399–416 (2005).
26. Percival, M. Floral ecology of coastal scrub in southeast Jamaica. *Biotropica* **2**, 104–129 (1974).
27. Montero, A. C. *The ecology of three pollination networks*. Master's thesis, Aarhus Univ, Aarhus, Denmark (2005).

28. Philipp, M., Böcher, J., R. Siegismund, H. & R. Nielsen, L. Structure of a plant-pollinator network on a pahoehoe lava desert of the galápagos islands. *Ecography* **29**, 531–540 (2006).

29. Lundgren, R. & Olesen, J. M. The dense and highly connected world of Greenland's plants and their pollinators. *Arctic, Antarctic, Alp. Res.* **37**, 514–520 (2005).

30. Bundgaard, M. *Tidslig og rumlig variation i et plantebestøvernetværk, Specialerapport*. Ph.D. thesis, Univ of Aarhus, Aarhus, Denmark (2003).

31. Dupont, Y. L. & Olesen, J. M. Ecological modules and roles of species in heathland plant-insect flower visitor networks. *J. Animal Ecol.* **78**, 346–353 (2009).

32. Bek, S. *A pollination network from a Danish forest meadow*. Master's thesis, Aarhus University, Aarhus, Denmark (2006).

33. Stald, L. *Struktur og dynamik i rum og tid af et bestøvningsnetværk på Tenerife, De Kanariske Øer*. Master's thesis, University of Aarhus, Denmark (2003).

34. Witt, P. BSc thesis, University of Aarhus, Aarhus, Denmark (1998).

35. Bartomeus, I., Vilà, M. & Santamaría, L. Contrasting effects of invasive plants in plant-pollinator networks. *Oecologia* **155**, 761–770 (2008).

36. Bezerra, E. L., Machado, I. C. & Mello, M. A. Pollination networks of oil-flowers: a tiny world within the smallest of all worlds. *J. Animal Ecol.* **78**, 1096–1101 (2009).

37. Abreu, C. R. & Vieira, M. F. Os beija-flores e seus recursos florais em um fragmento florestal de Viçosa, sudeste brasileiro. *Lundiana* **5**, 129–134 (2004).

38. del Coro Arizmendi, M. & Ornelas, J. F. Hummingbirds and their floral resources in a tropical dry forest in mexico. *Biotropica* **22**, 172–180 (1990).

39. Ingversen, T. T. *Plant-pollinator interactions on Jamaica and Dominica: The centrality, asymmetry and modularity of networks*. Master's thesis, Univ of Aarhus, Aarhus, Denmark (2006).

40. Vázquez, D. P. & Simberloff, D. Indirect effects of an introduced ungulate on pollination and plant reproduction. *Ecol. Monogr.* **74**, 281–308 (2004).

41. Kaiser-Bunbury, C. N., Muff, S., Memmott, J., Müller, C. B. & Caflisch, A. The robustness of pollination networks to the loss of species and interactions: a quantitative approach incorporating pollinator behaviour. *Ecol. Lett.* **13**, 442–452 (2010).

42. Kaiser-Bunbury, C. N., Vázquez, D. P., Stang, M. & Ghazoul, J. Determinants of the microstructure of plant–pollinator networks. *Ecology* **95**, 3314–3324 (2014).

43. Vizentin-Bugoni, J. *et al.* Influences of sampling effort on detected patterns and structuring processes of a Neotropical plant-hummingbird network. *J. Animal Ecol.* **85**, 262–272 (2016).

44. Lasprilla, L. R. *Interações planta/beija-flor em três comunidades vegetais da parte sul do Parque Nacional Natural Chiribiquete, Amazonas*. Ph.D. thesis, Colômbia (2003).

45. Baird, J. W. The selection and use of fruit by birds in an eastern forest. *The Wilson Bull.* 63–73 (1980).

46. Beehler, B. Frugivory and polygamy in birds of paradise. *The Auk* **100**, 1–12 (1983).

47. Carlo, T. A., Collazo, J. A. & Groom, M. J. Avian fruit preferences across a Puerto Rican forested landscape: pattern consistency and implications for seed removal. *Oecologia* **134**, 119–131 (2003).

48. Crome, F. H. J. The ecology of fruit pigeons in tropical Northern Queensland. *Wildl. Res.* **2**, 155–185 (1975).

49. Frost, P. G. H. Fruit-frugivore interactions in a South African coastal dune forest. *Acta XVII Congr. Int. Ornithol.* **2**, 1179–1184 (1980).

50. Galetti, M. & Pizo, M. A. Fruit eating by birds in a forest fragment in southeastern brazil. *Ararajuba* **4**, 71–79 (1996).

51. Snow, B. K. & Snow, D. W. The feeding ecology of tanagers and honeycreepers in Trinidad. *The Auk* **88**, 291–322 (1971).

52. Snow, B. K. & Snow, D. W. *Birds and berries: A study of an ecological interaction* (Calton, England, 1988).

53. Hamann, A. & Curio, E. Interactions among frugivores and fleshy fruit trees in a Philippine submontane rainforest. *Conserv. Biol.* **13**, 766–773 (1999).

54. Jordano, P. El ciclo anual de los paseriformes frugívoros en el matorral mediterráneo del sur de España: importancia de su invernada y variaciones interanuales. *Ardeola* **32**, 69–94 (1985).

55. Lambert, F. Fig-eating by birds in a Malaysian lowland rain forest. *J. Trop. Ecol.* **5**, 401–412 (1989).

56. Tutin, C. E., Ham, R. M., White, L. J. & Harrison, M. J. The primate community of the Lopé Reserve, Gabon: diets, responses to fruit scarcity, and effects on biomass. *Am. J. Primatol.* **42**, 1–24 (1997).

57. Mack, A. L. & Wright, D. D. Notes on occurrence and feeding of birds at Crater Mountain biological research station, Papua New Guinea. *Emu* **96**, 89–101 (1996).

58. Wheelwright, N. T., Haber, W. A., Murray, K. G. & Guindon, C. Tropical fruit-eating birds and their food plants: a survey of a Costa Rican lower montane forest. *Biotropica* **16**, 173–192 (1984).

59. Silva, W., De Marco Júnior, P., Hasui, E. & Gomes, V. Patterns of Fruit-Frugivore Interactions in Two Atlantic Forest Bird Communities of South-eastern Brazil: Implications for Conservation. *Seed dispersal frugivory: ecology, evolution conservation* 423–435 (2002).

60. Noma, N. Annual fluctuations of sapfruits production and synchronization within and inter species in a warm temperate forest on Yakushima Island. *Tropics* **6**, 441—449 (1997).

61. Guitián, J. *Relaciones entre los frutos y los passeriformes en un bosque montano de la cordillera cantábrica occidental* (Univ. Santiago, Spain, 1983).

62. Heleno, R. H., Ramos, J. A. & Memmott, J. Integration of exotic seeds into an Azorean seed dispersal network. *Biol. Invasions* **15**, 1143–1154 (2013).

63. Poulin, B., Wright, S. J., Lefebvre, G. & Calderon, O. Interspecific synchrony and asynchrony in the fruiting phenologies of congeneric bird-dispersed plants in Panama. *J. Trop. Ecol.* **15**, 213—227 (1999).

64. Davidson, D. W. *Symbiosis of ants with Cecropia as a function of light regime* (Ant - Plant Interactions, Oxford University Press, 1991).

65. Fonseca, C. R. & Ganade, G. Asymmetries, Compartments and Null Interactions in an Amazonian Ant-Plant Community. *J. Animal Ecol.* 339–347 (1996).

66. Blüthgen, N., E. Stork, N. & Fiedler, K. Bottom-up control and co-occurrence in complex communities: honeydew and nectar determine a rainforest ant mosaic. *Oikos* **106**, 344–358 (2004).
